# A Regenerative Approach to Canine Osteoarthritis Using Allogeneic, Adipose-Derived Mesenchymal Stem Cells. Safety Results of a Long-Term Follow-Up

**DOI:** 10.3389/fvets.2020.00510

**Published:** 2020-08-13

**Authors:** Éva Kriston-Pál, Lajos Haracska, Paul Cooper, Endre Kiss-Tóth, Valéria Szukacsov, Éva Monostori

**Affiliations:** ^1^Stem CellX Limited, Szeged, Hungary; ^2^Institute of Genetics, Biological Research Centre, Szeged, Hungary; ^3^Assentra Limited, Chelmsford, United Kingdom; ^4^University of Sheffield, Department of Infection, Immunity & Cardiovascular Disease, Sheffield, United Kingdom

**Keywords:** dogs, osteoarthritis, mesenchymal stem cell, therapy, safety of therapy, long-term follow-up

## Abstract

Mesenchymal stem cells (MSC) are emerging as an effective therapeutic tool in treating canine osteoarthritis (OA). In this report, we focused on the questions of whether MSC transplantation has long-term beneficial effects for the improvement in motion and also evaluated the safety of MSC injection. Visceral adipose tissue, a surgical waste obtained during routine ovariectomy served as a source of allogeneic MSCs and used to treat OA. Altogether, fifty-eight dogs were transplanted in the study suffering from OA in the elbow (42 animals), hip (5), knee (8), ankle (2), and hock (1). The effect of MSC transplantation was evaluated by the degree of lameness at a 4-5-years follow-up period based on the owners' subjective observations. The results showed that 83% of the OA patients improved or retained improvement in lameness. Clinical safety of the treatment was assessed by evaluating the coincidence of tumors or other diseases and other adverse reactions (such as local inflammation) after MSC cell therapy. Two incidences of local inflammation for <1 week at the site of injection were reported. No other adverse reactions were detected post-treatment. Sixteen dogs died during the study, 4 due to cancer and 12 due to other diseases, diagnosed by veterinarians. Overall, our survey suggests that MSC transplantation has long-term beneficial effects in reducing lameness. Moreover, no enrichment in a specific cause of death was observed in the transplanted animals, compared to reported literature. Our data suggest that MSC treatment could be an effective and safe long-term therapy for canine OA.

## Introduction

Osteoarthritis (OA), in which the integrity of joint cartilage is disrupted, is one of the most prevalent degenerative diseases, both in dogs and humans (1, 2). Due to the low self-regeneration capacity of the cartilage matrix, the disease is irreversible, thus, the quality of life of the affected animal is expected to decrease gradually (3). In the United Kingdom alone, 200,000 new cases of OA are diagnosed in dogs annually, with both external (injury, obesity, age) and genetic factors contributing (1, 4, 5). At present, the disease is treated symptomatically by regularly administering non-steroidal anti-inflammatory drugs or by repeated injections of hyaluronic acid (6, 7). Surgical solutions such as arthrodesis or excision arthroplasty (e.g., total joint replacement) are also used in more severe cases (8). However, these are invasive procedures, which may also carry risks of complications such as infections, instability, or periprosthetic fracture (2). In view of the above, the development of a safe and long-term regenerative treatment for OA is highly sought after.

MSCs are excellent candidates for this purpose with a rapidly expanding published literature demonstrating the effectiveness of these cells in tissue regeneration in various diseases (9). An increasing body of evidence now demonstrates that MSCs administered directly into the joint cavity can reduce the chronic pain caused by cartilage degeneration, can induce hyaline cartilage formation, and the treated dogs can live a highly improved quality of life (10, 11). Though MSCs are present in all tissues, adipose tissue is a major source of therapeutic MSCs due to easy access and their high numbers (12).

Besides being able to differentiate into chondrocytes, MSCs produce bioactive molecules, some of which have chondroprotective activity, while others are immunosuppressive and/or anti-inflammatory, thus enabling the safe injection of MSCs into recipients (13). However, the immunological status of the microenvironment is also critical since differentiation of MSCs is inhibited by inflammation (14).

We previously reported that intra-articular injection of allogeneic, adipose-derived MSCs, combined with hyaluronic acid induced a significant improvement in clinical signs of lameness, lasting for 1 year in dogs suffering from elbow OA (11) and now provide data on the long-term (>4 years) health status of the treated animals. To the best of our knowledge, the current report is the first such long-term survey published. We hypothesized that MSCs are effective in reducing lameness and improving the quality of life of animals and that local MSC transplantation is not associated with an increased prevalence of other diseases or malignancies.

## Methods

### Preparation of Visceral Adipose Tissue (AT) Mesenchymal Stem Cells (MSCs) for Therapy

MSCs were extracted from visceral adipose tissue. Adipose tissue was obtained as surgical waste during routine ovariectomy of healthy, female, mixed-breed dogs (age: between 7 months and 3 years). Donor dogs underwent all routine vaccinations and were regularly surveyed by veterinarians. Stromal vascular fraction (SVF) and then AT-MSC cultures were generated as previously described (11). The purity and differentiation ability of the resulting MSCs were characterized as described previously (11).

### Preparation of AT-MSCs for Therapy

Passage 2 AT-MSCs from two different adipose tissue donors were thawed, cultured for 3 days, mixed, and suspended in 0.5% sodium hyaluronate (TRB Chemedica International S.A. Geneva, Switzerland). Adipose tissue-derived MSCs (12 × 10^6^ ± 3.2 × 10^6^ cells/injection) were transported to the veterinarian clinics in syringes at 4–10°C and injected intra-articularly, within 24 h of dispensing, in a final volume of 1 mL.

### Patient Selection and Assessment of the Therapy

This study was approved by the National Scientific Ethical Committee of the National Food Chain Safety Office. All the dog owners signed an informed consent authorizing treatment and were informed of the possible risks of joint injections and potential complications of the procedure.

Fifty-eight dogs suffering from medium or severe osteoarthritis of various joints were included in the study. Severity of OA was evaluated by the participating veterinarians according to a modified method of Black et al. (15) as medium (intermittent but frequent lameness treated with NSAID medication or hyaluronic acid injection) or severe (no cartilage based on the X-ray analysis, continuous pain and lameness, reduction of joint movement, and presence of joint stiffness). The inclusion criteria were recurrent lameness and pain attributed to OA after conventional treatment of dogs (non-steroidal anti-inflammatory drugs, intra-articular injection of hyaluronic acid, arthroscopy, or traditional surgery focusing on debridement and removal of debris from the synovial cavity). Only those dogs were classed as “improved” that did not require further conventional treatment due to their OA during the study period.

The health status, including degree of lameness of the treated dogs was evaluated by the owners using a questionnaire modified from Black et al. (15) (Supplementary Table 1), with an occasional participation of the local veterinarian during the first year of follow-up. Further follow-up of up-to 5 years was based on the owners' subjective observations, reporting the degree and frequency of lameness and the possible necessity of pain relief medication since the MSC treatment in personal telephone interviews. The development of other diseases or cause of death was diagnosed by veterinarians during the 5 year follow-up period. The owner-assessed efficacy of the MSC transplantation in OA was evaluated to provide long-term, supportive information on the clinical outcomes following this treatment.

There were no exclusion criteria except for pre-existing, diagnosed cancer.

## Results

Overall, fifty-five out of 58 transplanted animals were available for the long-term follow-up. Forty-two animals suffered from elbow OA (Table 1 and Supplementary Table 2); all suffering from elbow dysplasia, except two dogs (aged 12 and 13 years) which had developed cartilage degeneration probably due to their age. Eleven dogs received MSCs into both elbows, while 31 animals obtained transplant into one joint (Supplementary Table 2). Thirty-nine dogs were available for the long-term follow-up, by the end of which the average age of the dogs in the study increased from 3 to 7.3 years.

**Table 1:** Summary of long-term follow-up after MSC transplantation.

**Site of OA**	**No. of animals included/No. of animals evaluated**	**Range of age (average) at the time of MSC injection in years**	**Average age at the time of follow-up (years)**	**Results at 2.5, 4, or 5-year follow-up**		**Joint or spinal diseases other than the transplanted joint**	**Number and cause of death**	
				**Improved or sporadic lameness and/or medication during weather fronts or extreme activity**	**No improvement continuous medication for other joint/spinal problems or death**		**Other than cancer**	**Cancer**
Elbow OA	39^*^/31^**^	0.6–10 (3)	7.3	26 (84%)	5	9	8	3
Hip OA	5/4^&&^	1.5–8 (5.5)	9.7	3	1	2	1	1
Knee OA, dislocation, ligament tear	8/6^&&^	3–10 (5.6)	10	5 1	1	3	3	-
Hock OA	1	0.8	5	1	-	-	-	-
Ankle OA	2	0.4–1.5 (0.96)	5.5	2	-	-	-	-
Total	55^&^/44^&&^	0.4–10 (3.2)	7.5	37 (84%)^#^	7 (16%)	14	12	4

*The condition (lameness, usage of drugs) of the transplanted joint of the same animal was followed up for 2.5, 4, or 5 years depending on the date of transplantation*.

# *% = (Improved animal number: number of animals in the study) × 100*.

* *6 dogs died within 1 year of the survey*.

** *3 of them died just before the end of the survey, but their condition was evaluated till death*.

& *One dog died within 1 year of the survey and 3 died later but before the end of the follow-up*.

&& *One dog who died before the end of the survey was evaluated till death*.

Sixteen dogs that received the MSC treatment into other joints [knee (8), hip (5), ankle (2), and hock (1)] (one joint in one animal, see Supplementary Table 2) were also evaluated in the long-term follow-up (Table 1). The average age was 4.8 years at the time of transplantation, increasing to 9 years by the end of the study period.

Thirty-seven dogs out of 44 (84%), were reported by the owners to have reduced lameness and an improved quality of life until the end of the follow-up period or the death of the treated animals. Several dogs improved, and although suffered from other joint problems did not require medication. Seven dogs diagnosed with other joint or spinal problems besides elbow OA and hence receiving continuous medication were also included into the survey period of elbow OA (Table 1).

Two of 58 dogs showed a short-term local inflammation after MSC transplantation. This inflammatory reaction resolved within a week and did not affect the long-term improvement in the quality of the dogs' life (data not shown).

Sixteen dogs between the ages of 7 and 13 died during the follow-up period, four of them of cancer (between the ages of 11 and 13) and 12 due to various other diseases (aged 7–12 years) (Table 1). Cases of cancer included: melanoma, epithelial cancer, metastatic cancer, spleen-liver cancer (a single case for each tumor-type). Other co-morbidities reported by the owners included: epilepsy, pancreatitis, volvulus, heart failure, ulcers, neurological, and musculo-skeletal problems. None of these syndromes were reported more than twice, suggesting that they may not be associated with the MSC treatment.

## Discussion

The aim of this study was to explore whether local intra-articular transplantation of MSC had long-term beneficial effects on lameness and hence improving quality of life of the transplanted animals and whether it caused any serious adverse effects or correlated with an increased prevalence of other diseases.

Our data presented in this paper suggest that MSC transplantation results in improvement of motion. In the current study, 60% of the treated animals belonged to 3 breeds: Golden Retriever (9), Labrador Retriever (16), and German Shepherd (8). This is consistent with these breeds being generally recognized as at high risk of OA (1). The sex of the animals did not correlate with either the coincidence of OA or the death occurring subsequently to MSC injection. Our results presented here are in line with the literature suggesting a beneficial effect of MSC transplantation in OA (11, 16).

In the cohorts included in the long-term survey, most dogs suffered from elbow OA (42 dogs out of 58) of which 40 were diagnosed with elbow dysplasia, hence elbow dysplasia was accepted as the main cause of osteoarthritis in the elbow. Eighty four percentage of the animals maintained an improved condition (no lameness/no medication or sporadic lameness and/or medication during wet weather or with extreme activity) by the subjective assessment of the owners.

Osteoarthritis cases in joints other than the elbow (knee, hip, ankle, and hock) were also evaluated. Eighty four percentage of the evaluated dogs retained their improved condition after the 4-5-year follow-up. It should be noted that the dogs in this mixed group were of higher age than those in the elbow group, and many patients suffered from joint or spinal problems in addition to the treated joint.

MSC transplantation does not appear to be associated with an increase in malignancies or other diseases, and no other adverse effects emerged due to MSC injection. These findings are underpinned by: (1) the published literature that supports that MSC is not a tumorigenic cell type (17, 18); (2) published findings that local injection into the joint results in the adherence of MSCs to the damaged cartilage (19) with no reported evidence for their migration outside of the treated joint. Sixteen dogs out of 58 transplanted animals died during the long-term follow-up: four due to cancer in old age (11–13 years) and 12 from other different causes.

This rate, in spite of the small number of cases overall, is comparable to large-scale disease prevalence statistics in the USA (5.3%) thus does not indicate an association between prevalence of cancer and MSC transplantation (20). Other studies report a prevalence of 3.4–8.63% for melanoma (in dogs the age average was 7, 5 years) in Switzerland and Brazil and 3.4% for internal organ tumors in India over a period of 10 years (21–23). Published literature demonstrates a 4.9% prevalence of death due to heart failure in dogs with a median of 9.9 years in the UK (24), whilst our study reports 3.6%. Of note, the dog population included in our follow-up study is comprised of older dogs, thus, the number of deaths due to tumors is expected to be higher than that in the whole population. In our study, however, the average age of the dogs that died due to tumors was 12.2 years, while in a previous report from the USA involving golden retrievers it was 9.83 years (25).

The potential use of allogeneic MSCs for the treatment of various diseases has also been piloted, including humans and horses, for instance, for indications such as cardiac damage post-myocardial infarction and wound healing (26, 27). However, whilst long-term safety data is still very sparse, evidence is now emerging on a convincing safety profile for the use of allogeneic MSCs in cardiac regeneration (28) and autoimmune disease in man (29) as well as in osteoarthritis in horses (30). Our current report is in alignment with this broader literature and further highlights the potential for the use of allogeneic MSCs in a variety of diseases.

In summary, our data suggest that intra-articular injection of allogeneic MSCs may provide a long-term improvement in lameness secondary to OA, based on subjective reporting of the owners, without the risk of long-term adverse effects on health. Thus, we conclude that MSC treatment could be an effective and safe long-term therapy for canine OA.

Though the results overall suggest that MSC treatment is beneficial for dogs suffering from osteoarthritis, evaluation is limited by the fact that the improvement was subjectively compared to the initial state by owners. To overcome these limitations, a follow-up with rigorous orthopedic examination performed by veterinarians will be recommended.

## Data Availability Statement

All datasets generated for this study are included in the article/Supplementary Material.

## Ethics Statement

The animal study was reviewed and approved by Csongrád County Committee on the Food Chain and Animal Health, Government Office of Csongrád County, Rákóczi tér 1, Szeged 6722 Hungary. Written informed consent was obtained from the owners for the participation of their animals in this study.

## Author Contributions

EK-P: preparing MSCs for therapy. LH: organization of contacts with vets and owners. PC and EK-T: Evaluating results. VS: keeping contact and interviewing the owners. EM: leading the study. All authors: contributed to writing the manuscript.

## Conflict of Interest

EK-P was employed by company Stem CellX Europe Limited. PC and EK-T are shareholders of Stem CellX Europe Limited and PC is a shareholder of Assentra Limited. The remaining authors declare that the research was conducted in the absence of any commercial or financial relationships that could be construed as a potential conflict of interest.
