# Supplementary material for: A Regenerative Approach to Canine Osteoarthritis Using Allogeneic, Adipose-Derived Mesenchymal Stem Cells. Safety Results of a Long-Term Follow-Up
Source: Front Vet Sci. 2020 Aug 13;7:510. doi: 10.3389/fvets.2020.00510 (PMC7438407; doi:10.3389/fvets.2020.00510)
Supplement: Supplementary file 1 [file Table_1.DOCX]

|  | Cannot be detected | Intermittent | Persistent | Persistent – unable to bear weight | Can only walk with help | Unable to walk |
| --- | --- | --- | --- | --- | --- | --- |
| Lameness in walking | 1 | 2 | 3 | 4 | 5 | 6 |
| Lameness in trotting | 1 | 2 | 3 | 4 | 5 | 6 |
|  | No pain | | Mild pain – tries to pull up its leg | | Severe pain – pulls its leg back immediately | |
| Pain – to touch, pressure, movement | 1 | | 2 | | 3 | |
|  | No limitation | Pain at the end point only | | Pain before the end point | | Pain to movement of the joint |
| Range of motion | 1 | 2 | | 3 | | 4 |
|  | Normal activity | Shligthly stiff gait, only noticable on running | Stiff, dog has noticable difficulty walking or running | Very stiff, dog does not want to walk or run unless coaxed | | Dog does not want to walk, must be helped up, and will not run |
| Functional disabilities | 1 | 2 | 3 | 4 | | 5 |
|  | Not required | | Intermittently required | | Constant pain relief required | |
| Requirement of pain relief medication | 1 | | 2 | | 3 | |
|  | 12 months | 9 months | 6 months | 3 months | 1 month | |
| Asymptomatic period | 1 | 2 | 3 | 4 | 5 | |

Supplementary Table 1. Scoring table used by owners one year after the treatment (modified Black assessment table). During the 4-5-year follow-up, our questions focused on lameness in walking, requirement of pain relief medication, and general health.
