# Supplementary material for: A Regenerative Approach to Canine Osteoarthritis Using Allogeneic, Adipose-Derived Mesenchymal Stem Cells. Safety Results of a Long-Term Follow-Up
Source: Front Vet Sci. 2020 Aug 13;7:510. doi: 10.3389/fvets.2020.00510 (PMC7438407; doi:10.3389/fvets.2020.00510)
Supplement: Supplementary file 2 [file Table_2.DOCX]

| **Type of OA** | | **Sex** | **History Before MSC Injection** | | | | **Date of MSC Injection** | | **Age at the Date of MSC Injection** | | **Period of Follow-up** | | **Clinical Condition at Assessment** | **Treatment success/**  **failure/**  **at Assessment** | **Disease Other than OA** | **Age and Cause of Death (if known)** | |
| --- | --- | --- | --- | --- | --- | --- | --- | --- | --- | --- | --- | --- | --- | --- | --- | --- | --- |
|  |  |  | **Surgery** | | **HA**  **Injection** | |  |  |  |  |  |  |  |  |  |  |  |
| **ELBOW OA** | |  |  | |  | |  | |  | |  | |  |  |  |  | |
| ELBOW OA  GR | | male | + | | +(2x)  1 and 2 months effect | | Both elbows  01.12.2013 | | 22 months | | 5 years | | No lameness, no medication | + | Drug Resistant Epilepsy (DRE) | Euthanized at the age of 7 due to DRE | |
| ELBOW OA  GR | | male | + | | - | | Both elbows  08.27.2014 | | 10 months | | 5 years | | Sporadic lameness after long and intensive walk,  no medication | + | - | - | |
| ELBOW OA  Dogue de Bordeaux /Amstaff | | male | + | | - | | Both elbows  08.27.2014 | | 4 years | | 5 years | | No lameness, no medication | + | - | - | |
| ELBOW OA  GR | | male | + | | - | | One elbow  08.27.2014 | | 7 months | | 5 years | | Sporadic lameness after long and intensive walk,  no medication | + | - | - | |
| ELBOW OA  GS | | male | + | | -/? | | One elbow  11.26.2014 | | 12 years | | - | | - | NA | Melanoma, sarcoma metastatic | Euthanized at the age of 12 due to melanoma* | |
| ELBOW OA  Chow chow | | female | + | | +  Effect for 6 weeks | | Both elbows  11.26.2014 | | 8 years | | 5 years | | No lameness,  One year before the end of assessment no.2 sporadic Meloxidyl, Trocoxil medication. as prevention due to unknown indication | + | Cruciate ligament tear surgery, removal of implant | - | |
| ELBOW OA  LR | | male | + | | - | | Both elbows  12.03.2014 | | 3 years | | 5 years - | | Sporadic lameness after intensive activity, short periods of Meloxidyl medication | + | - | - | |
| ELBOW OA  HV | | female | + | | - | | Both elbows  10.01.2014 | | 1 year | | 5 years | | No lameness, no medication | + | - | - | |
| ELBOW OA  LR | | male | + | | - | | One elbow  12.03.2014 | | 3 years | | - | | - | NA | - | - | |
| ELBOW OA  Cane Corso | | male | + | | - | | One elbow  12.17.2014 | | 1 year | | 5 years | | Forelegs are not lame | NA | Hip dysplasia Medication Trocoxil | - | |
| ELBOW OA  LR | | male | +  Arthroscopy  no cartilage | | - | | One elbow  11.11.2014 | | 9 years | | 5 years | | The dog is 14 years old, unable to stand up likely due to old age | . | - | - | |
| ELBOW OA  Mixed breed | | male | + | | - | | Both elbows  12.17.2014/03.06.2015 | | 1 and 2 years | | 5 years | | No lameness in forelegs. Medication for hip dysplasia | NA | Hip dysplasia. Medication for hip dysplasia | - | |
| ELBOW OA  Mixed breed | | female | + | | - | | One elbow  12.17.2014 | | 7 months | | 5 years | | No lameness in forelegs Medication for hip dysplasia | NA | Hip dysplasia. Medication: Trocoxil. MSC TP into the hip joint did not help. | - | |
| ELBOW OA  GR | | female | + | | - | | Both elbows  01.19.2015 | | 3 years | | 4 years | | No lameness, no medication | + | - | - | |
| ELBOW OA  Cane Corso | | male | + | | - | | One elbow  01.19.2015 | | 3 years | | 4 years | | No lameness,  no medication except during weather fronts | + | Epilepsy | Died at age of 7 of epilepsy | |
| ELBOW OA  GS mix | | male | + | | - | | One elbow  01.19.2015 | | 5 years | | 4 years | | Occasional lameness, no medication | + | - | - | |
| ELBOW OA  LR | | male | + | | - | | One elbow  02.06.2015 | | 4 years | | 4 years | | No lameness, no medication | + | - | - | |
| ELBOW OA  LR | | female | + | | - | | One elbow  02.06.2015 | | 3 years | | 4 years | | No lameness, no medication | + | - | - | |
| ELBOW OA  LR | | male | + | | - | | One elbow  02.06.2015 | | 2 years | | 4 years | | No lameness, no medication | + | - | - | |
| ELBOW OA  LR | | male | + | | - | | One elbow  02.06.2015 | | 2 years | | 4 years | | No lameness,  no medication | + | Slipped disc | - | |
| ELBOW OA  English bulldog | | female | + | | - | | One elbow  02.13.2015 | | 7 months | | 4 years | | No lameness, no medication,  very active | + | - | - | |
| ELBOW OA  Rottweiler | | male | + | | - | | One elbow  02.13.2015 | | 4 years | | 4 years | | Well without medication prior to death. | +  till death due to other disease (pancreatitis) | Pancreatitis | Died at age of 9 of pancreatitis | |
| ELBOW OA  GS | | male | + | | +  No effect | | One elbow  02.27.2015 | | 13 years | | - | | - | NA | Epithelial cancer | Euthanized at the age of 13 due to epithelial cancer* | |
| ELBOW OA  Mixed breed | | male | + | | - | | Both elbows  02.27.2015 | | 8 years | | 4 years | | The hind legs are weak, but the forelegs work well. Due to old age, he moves slowly. | + | Severe hip dysplasia,  spondyle coalescence |  | |
| ELBOW OA  GR | | female | + | | - | | Both elbows  02.27.2015 | | 1 year | | 4 years | | Lameness during weather fronts, no medication | “ | Hip dysplasia | - | |
| ELBOW OA  Pyrenean Mountain Dog | | male | + | | - | | One elbow  03.06.2015 | | 7 years | | - | | - | NA | volvulus | Died of volvulus at the age of 7* | |
| ELBOW OA  LR | | Not known | + | | - | | One elbow  03.06.2015 | | 3 years | | 4 years | | Improved after Assessment No.1  no lameness, no medication. | + | - | - | |
| ELBOW OA  Sharpei | | male | + | | - | | One elbow  03.13.2015 | | 6 months | | - | | - | NA | - | - | |
| ELBOW OA  GR | | male | - | | - | | One elbow  03.13.2015 | | 5 years | | 4 years | | No lameness, no medication | + | - | - | |
| ELBOW OA  GR | | male | + | | +  No effect | | One elbow  03.13.2015 | | 1.5 years | | 4 years | | For three years no lameness, no medication. During the last year Rimadyl for occasional lameness. | + | - | - | |
| ELBOW OA  Puli | | female | + | | - | | One elbow  04.03.2015 | | 8 years | | - | | - | NA | - | - | |
| ELBOW OA  GS | | male | - | | - | | Both elbows  04.03.2015 | | 12 years | | - | | - | NA | Lumbar spine problems,  hip dysplasia | Died at the age of 12 of heart failure* | |
| ELBOW OA  LR | | female | + (2x) | | - | | One elbow  04.03.2015 | | 1.5 years | | 4 years | | No lameness, no medication | + | Hind leg problems, surgery | - | |
| ELBOW OA  LR | | female | + | | - | | One elbow  04.03.2015 | | 10 months | | 4 years | | No lameness except during strong weather fronts. No medication. Hunting in autumn and winter, wild up to 3-400 games a day | + | - | - | |
| ELBOW OA  GS | | male | - | | - | | One elbow  04.10.2015 | | 5 years | | 4 years | | 9 years old. Healthy condition, working dog. Lameness after extensive activity. Occasional medication (Rimadyl). | + | - | - | |
| ELBOW OA  GS | | male | + | | - | | One elbow  04.10.2015 | | 9 years | | - | | - | NA | HD prosthesis 2 months before trans-plantation, surgery if hidden testis at the time of TP | Died due to unknown reason at the age of 9* | |
| ELBOW OA  GR | | female | + | | - | | One elbow  04.10.2015 | | 2 years | | 4 years | | Occasional lameness after extensive activity.  No medication. | + |  |  | |
| ELBOW OA  Swiss White Shepherd | | male | + | | - | | One elbow  04.10.2015 | | 8 months | | - | | - | + | - | - | |
| ELBOW OA  LR | | female | + | | - | | Both elbows  04.10.2015 | | 7 years | | 4 years | | There is no pain in the elbows. Medication: pain killer for [spinal disc herniation](https://dictzone.com/angol-magyar-szotar/spinal%20disc%20herniation). | NA | [Spinal disc herniation](https://dictzone.com/angol-magyar-szotar/spinal%20disc%20herniation), central ventricle dilatation, stomach ulcer Medication: pain killer for [spinal disc herniation](https://dictzone.com/angol-magyar-szotar/spinal%20disc%20herniation). | Died in 2017 at the age of 11during anesthesia for operation of stomach ulcer. | |
| ELBOW OA  LR | | female | + | | - | | Both elbows  04.10.2015 | | 10 years | | - | | - | NA | Metastatic tumor, spasticity | Euthanized due to metastatic tumor at the age of 13 | |
| ELBOW OA  Giant Schnauzer | | female | + | | - | | One elbow  04.03.2015 | | 8 years | | - | | - | NA | - | Died due to unknown reason at the age of 8* | |
| ELBOW OA  LR | | Not known | + | | - | | One elbow  05.27.2017 | | 3 years | | 2.5 years | | No lameness, no medication | + | - | - | |
| **HIP OA** | |  |  | |  | |  | |  | |  | |  |  |  |  | |
| HIP OA  GS | | male | - | | - | | One hip  12.03.2014 | | 7 years | | 5 years | | Died of cancer at the age of 11. | +  till death | Tumor | Died of spleen-liver cancer at the age of 11. | |
| HIP OA  Bernese Mountain Dog | | male | - | | - | | One hip  02.13.2015 | | 1.5 years | | 4 years | | 8 years old, which is an old age for this breed. Lameness, and medication twice a year | + | - | - | |
| HIP OA  LR | | male | - | | - | | One hip    02.13.2015 | | 8 years | | 4 years | | - | NA | Epilepsy-like seizures, long-term Trocoxil till the 6^th^ month of TP. | Died at the age of 11 of pancreatitis | |
| HIP OA  Border Collie | | male | - | | - | | One hip  03.06.2015 | | 6 years | | 4 years | | Femoral head resection in 2018. Since then, the dog is well. | + | - | - | |
| HIP OA  GS | | male | - | | - | | One hip  04.03.2015 | | 5 years | | 4 years | | The hip is in bad condition, femoral head resection will be required. | - | - | - | |
| **KNEE OA** | |  |  | |  | |  | |  | |  | |  |  |  |  | |
| KNEE OA  Belgian Shepherd | | female | - | | - | | One knee  12.03.2014 | | 10 years | | 5 years | | No medication for 2 years. At the age of 12, her condition started declining, now she gets NSAID (last four months of the end of the survey period) | +  for 3 years after MSC injection | Spine problems, liver cancer (14 years old) | - | |
| KNEE OA  GS | | female | - | | - | | One knee  03.06.2015 | | 6 years | | 4 years | | She does not load the transplanted leg equally but it did not deteriorate during the years. | + | - | - | |
| KNEE OA  Australian Shepherd | | female | + | | - | | One knee  03.06.2015 | | 3 years | | 4 years | | The transplanted knee is well.  During the last 5 months of the 2^nd^ assessment, the spinal cord pressure intensified, therefore received steroid. | + | Undiagnosed  shoulder problems | - | |
| KNEE OA  Doberman | | male | . | | . | | One knee  03.13.2015 | | 7 years | | - | | - | NA | - | Suddenly died of heart failure at the age of 11 years | |
| KNEE OA  Irish Wolf Dog | | female | - | | - | | One knee  04.03.2015 | | 4 years | | 5 years | | There was no improvement in the condition. At the age of 7 she was euthanized. | -  till death | Hip dysplasia,  vertebral disk surgery, neurological problem. | Euthanized at the age of 7 due to neurological problem. | |
| KNEE OA  Caucasian Shepherd | | female | - | | - | | One knee  04.10.2015 | | 7 years | | - | | - | NA | multiple musculoskelet-al problems | Euthanized at age of 7 years, 5 months after TP due to multiple musculo-skeletal problems* | |
| Knee dislocation  Pomeranian | | female | + | | - | | One knee  02.27.2014 | | 6 years | | 5 years | | No lameness, no medication. | + | - | - | |
| Knee ligament tear  LR | | male | + | | - | | One knee  12.20.2015 | | 3 years | | 5 years | | Occasional lameness.  Medication 2-3x/year as a cure.  Otherwise active dog. | + | - | - | |
| **ANKLE OA** | |  |  | |  | |  | |  | |  | |  |  |  |  | |
| Ankle OA  Rottweiler | | female | +  Removing implantatum | | - | | One ankle  11.26.2014 | | 5 months | | 5 years | | No lameness, no medication Occasional ankle swelling. |  | - | - | |
| Ankle OA  Border Collie | | male | + | | - | | One ankle  02.06.2015 | | 1.5 years | | 4 years | | No lameness, no medication.  Occasional ankle swelling without pain. | + | - | - | |
| **HOCK OA** |  | | |  | |  | |  | |  | |  | | | | |  |
| Hock OA  GR | | female | ? | | - | | One hock  02.13.2015 | | 10 months | | 4 years | | No lameness, no medication.  High activity. | + | - | - | |

**Supplementary Table 2.**  *Results of therapy with mesenchymal stem cells of fifty-eight dogs suffering from osteoarthritis*

GR: Golden Retriever; LR: Labrador Retriever; GS: German Shepherd; HV: Hungarian Vizsla

OA: osteoarthritis; HA: intra-articular hyaluronic acid, TP: MSC transplantation

* Died within one year of transplantation due to cancer or other disease

No asterisk in row “**age and cause of death”**: died before the 4-5-year follow-up due to cancer or other disease

Assessment: treatment success: +, treatment failure: -, NA: not applicable
